# The New Era of Canine Science: Reshaping Our Relationships With Dogs

**DOI:** 10.3389/fvets.2021.675782

**Published:** 2021-07-15

**Authors:** Evan L. MacLean, Aubrey Fine, Harold Herzog, Eric Strauss, Mia L. Cobb

**Affiliations:** ^1^School of Anthropology, University of Arizona, Tucson, AZ, United States; ^2^College of Veterinary Medicine, University of Arizona, Tucson, AZ, United States; ^3^Cognitive Science, University of Arizona, Tucson, AZ, United States; ^4^California State Polytechnic University, Pomona, CA, United States; ^5^Department of Psychology, Western Carolina University, Cullowhee, NC, United States; ^6^Center for Urban Resilience, Loyola Marymount University, Los Angeles, CA, United States; ^7^Animal Welfare Science Centre, Faculty of Veterinary and Agricultural Sciences, The University of Melbourne, Melbourne, VIC, Australia

**Keywords:** canine science, dog, animal welfare, human-animal interaction, science communication, funding, sustainability

## Abstract

Canine science is rapidly maturing into an interdisciplinary and highly impactful field with great potential for both basic and translational research. The articles in this *Frontiers* Research Topic, *Our Canine Connection: The History, Benefits and Future of Human-Dog Interactions*, arise from two meetings sponsored by the Wallis Annenberg PetSpace Leadership Institute, which convened experts from diverse areas of canine science to assess the state of the field and challenges and opportunities for its future. In this final *Perspective* paper, we identify a set of overarching themes that will be critical for a productive and sustainable future in canine science. We explore the roles of dog welfare, science communication, and research funding, with an emphasis on developing approaches that benefit people and dogs, alike.

Dogs have played important roles in the lives of humans for millennia (1, 2). However, throughout much of scientific history they have been dismissed as an artificial species with little to contribute to our understanding of the natural world, or our place within it. During the last two decades, this sentiment has changed dramatically; canine science is rapidly maturing into an established, impactful, and highly interdisciplinary field (Figure 1). Canine scientists, who previously occupied relatively marginalized roles in academic research, are increasingly being hired at major research universities, and centers devoted to the study of dogs and their interactions with humans are proliferating around the world. The factors underlying dogs' newfound popularity in science are diverse and include (1) increased interest in understanding dog origins, behavior, and cognition; (2) diversification in our approaches to research with non-human animals; (3) recognition of dogs' value as a unique biological model with relevance for humans; and (4) growth in research on the nature and consequences of dog-human interactions, in their myriad forms, from working dog performance to displaced canines living in shelters.

**Figure 1:**
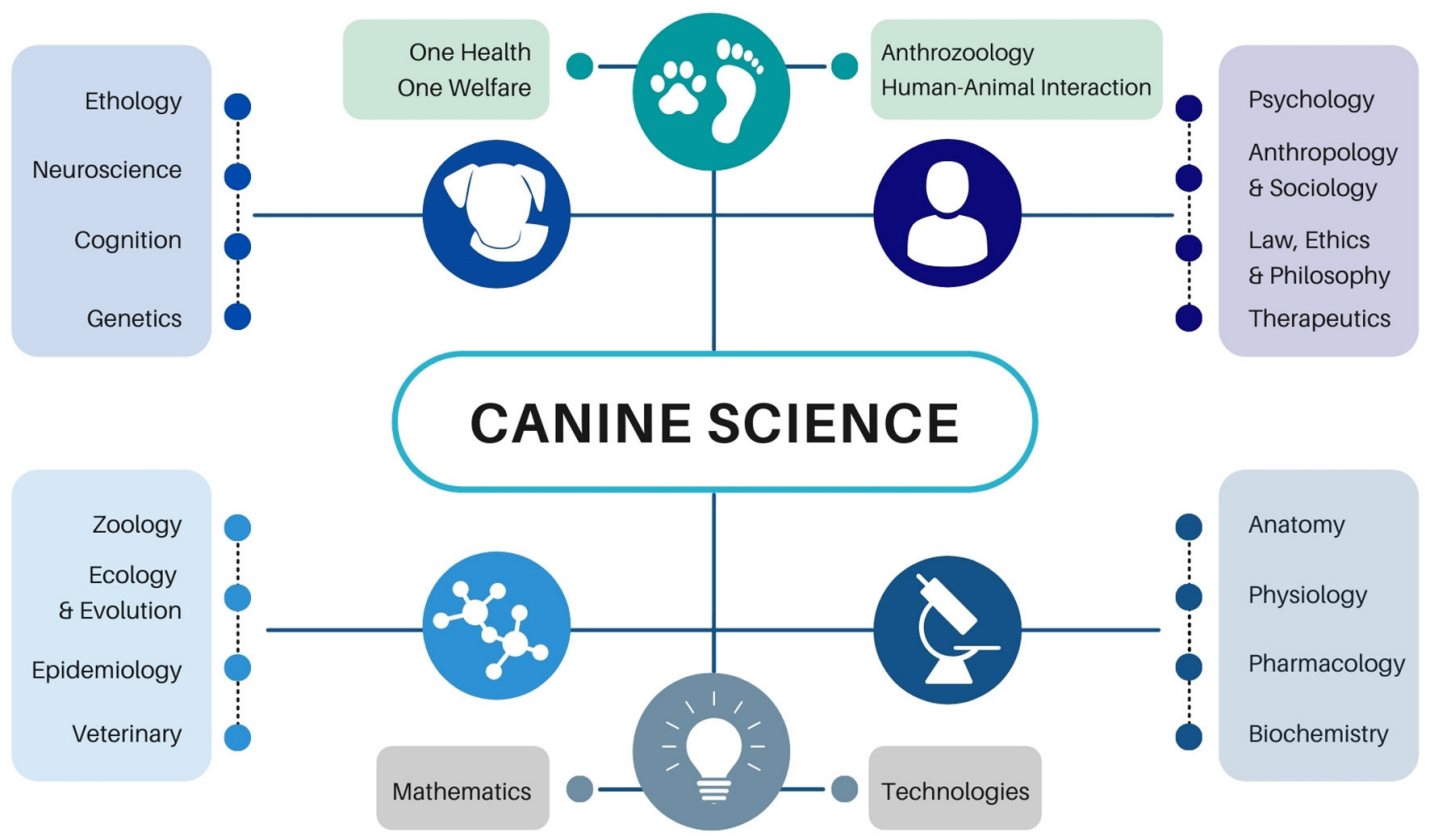
Canine science is an interdisciplinary field with connections to other traditional and emerging areas of research. The specific fields shown overlap in ways not depicted here and are not an exhaustive list of disciplines contributing to canine science. Rather, they are included as examples of the diversity of scholarship in canine science.

This Perspective represents the final article in a collection of manuscripts arising from two workshops sponsored by the Wallis Annenberg PetSpace Leadership Institute. Leadership Fellows from around the world gathered in 2017 and 2020 to discuss the state of research and future directions in canine science. The individual articles in this collection provide a detailed treatment of key topics discussed at these events. In this final article, we identify a set of overarching challenges that emerge from this work and identify priorities and opportunities for the future of canine science.

The rise of canine science has benefited substantially from public interest and participation in the research process. Unlike many research studies, which unfold quietly in the ivory towers of research universities, the new era of canine science is intentionally public facing. The dogs being studied are not laboratory animals, bred and housed for research purposes, but rather are companions living in private homes, or assisting humans in capacities ranging from assistance for people with disabilities, to medical and explosives detection. Campus-based research laboratories have opened their doors to members of the public who bring their dogs to participate in problem-solving tasks, social interactions, and sometimes even non-invasive neuroimaging studies. Increasingly, dog owners themselves play a significant role in the scientific process, serving as community scientists who contribute to the systematic gathering of data from the convenience of their homes.

This new research model in conjunction with emerging technologies, makes canine science a highly visible field that engages public stakeholders in unprecedented ways. From a scientific perspective, society has become the new laboratory, and in doing so, has facilitated research with dogs of a scope and scale that was heretofore unthinkable. As tens of thousands of dogs contribute to research on topics ranging from cognition and genetics (3, 4) to aging and human loneliness (5), canine science is entering the realm of “big data” and eclipsing many traditional research approaches. Importantly, these advances are occurring simultaneously across diverse fields of science, creating powerful new opportunities for consilience that will make canine science even more valuable in the years ahead. However, maturing this model toward a sustainable future that serves its diverse stakeholders—who include scientists, research funders, members of the public, and dogs themselves—will require careful navigation of key challenges related to dog welfare, science communication, and financial support (Figure 2).

**Figure 2:**
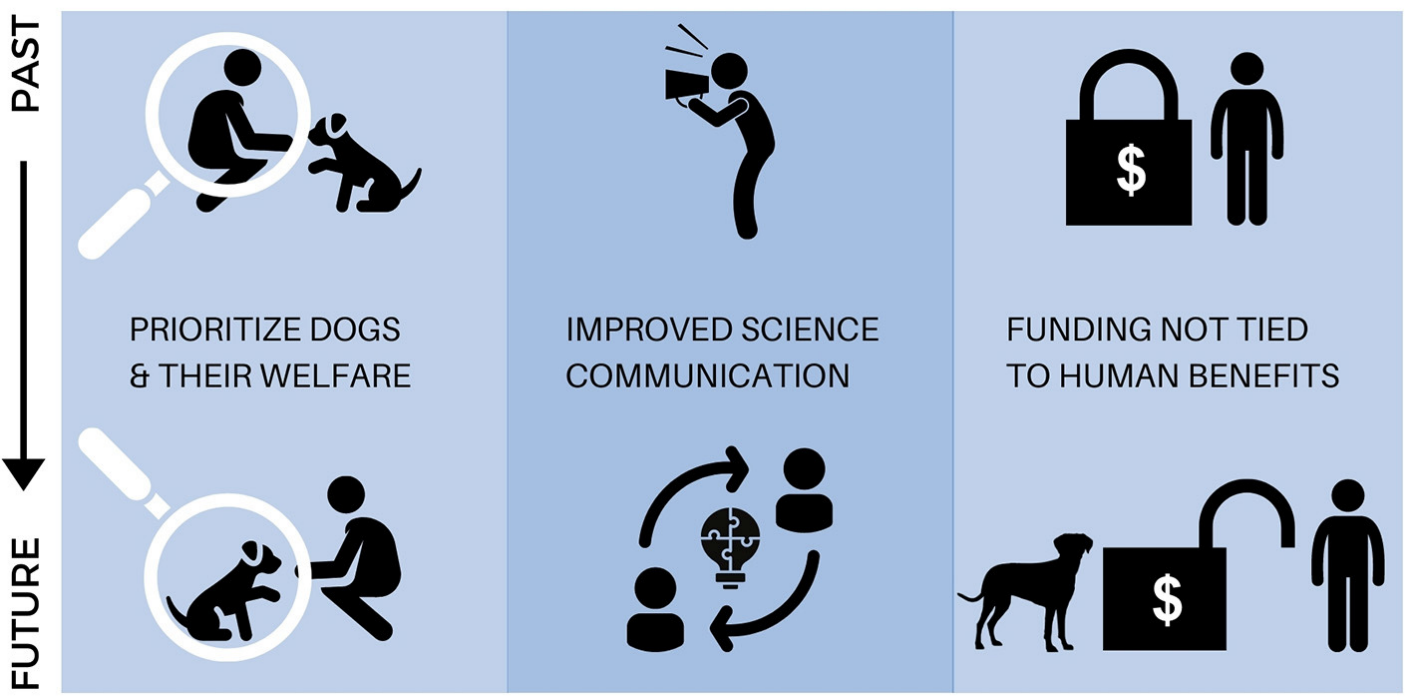
Visual summary of the key issues identified in this *Perspective*. A sustainable future in canine science will require (1) research approaches that prioritize and monitor the welfare of dogs, (2) improved science communication to avoid incorrect reporting of study results, and to translate research findings to meaningful change in practices relating to dogs, and (3) availability of research funding that is not tied exclusively to studying the possible benefits of dogs for humans.

## Dog Welfare

Globally, animal welfare has been linked to the public acceptability that underpins sustainable animal interactions and partnerships (6). Where human-animal interactions have failed to meet community expectations, practices and in some case entire industries, have been disrupted or ceased. Recent examples include whaling for profit and greyhound racing (6, 7). Science is not exempt from this necessity to meet with public expectations and the new era of canine science must place canine welfare at the forefront. Considering dogs as individuals and co-workers, rather than tools for work or subjects, reflects a community moral and ethical paradigm shift that is currently underway. Reimagining our relationship with domestic dogs in research will also help inform our treatment of other animals. In this way, studies of dogs and our interactions with them can serve as a pioneering new model for many areas of science.

As scientists advocate for the revision of community and industry practices with dogs in light of new evidence, we must apply the same criteria to the conduct of our research. This includes adjusting canine research and training methods to acknowledge the sentience of dogs, and the importance of the affective experience for dogs in both research and community settings (8–11). The discipline of animal welfare science has progressed rapidly over the last two decades, and we have many animal-based, welfare-outcome measures available to us (6, 11). Ensuring the well-being of the dogs we study will be as critical to ongoing social license to operate (i.e., community approval) for canine science as it is for working dog interests (12). Being transparent about the issues of animal consent and vulnerability, as well as offering animals agency with regard to their participation in science are valuable suggestions offered within this special issue. We encourage our colleagues to not just consider this paradigm shift, but to effect it through prioritizing and representing the dog's perspective and welfare in their research.

Although increasingly, researchers may include a single or limited set of canine stress measures in studies exploring dogs' potential benefits to humans, this approach alone does not fill the need for studies that prioritize an understanding of canine welfare as their central focus. Canine welfare should be considered not just as an emergent population-level measure (13) but rather with respect to the way in which it is experienced: from the perspectives of individual dogs. Commonly used statistical methods from human research, such as group-based trajectory analysis (14) may offer proven techniques that allow meaningful reporting on populations while reflecting the nuance of shared, sub-group patterns. Such approaches will better reflect individual differences, for example variations in canine personality, social support and relationship styles, as well as other significant factors. One impediment to robust measurement of animal welfare in canine science has been limited funding.

We believe that all granting bodies who fund exploration of the possible benefits to people from dogs should also fund and require the canine perspective to be robustly monitored and reported. Impediments to this work arise not from lack of researcher interest or access to dogs, but rather from challenges to securing funding that is independent from a focus on human health outcomes, or other tangible outcomes of work that dogs perform. To be able to optimize canine welfare, there is an urgent need for increased funding specifically to study the welfare of dogs, in all their diversity. The new era of canine science will identify what dogs need to thrive, propelling us toward a mutually sustainable partnership between people and dogs.

## Communication

One area that has not received much attention in relation to canine science is the way in which research findings are communicated outside the empirical literature. Fueled by media reports, interest in canine science and the impact of dogs on human health and well-being has grown substantially in the last 10 years. A survey by the Human-Animal Bond Research Institute found that 71% of pet owners were aware of studies demonstrating that pets improve mental and physical health. Some of these claims are justified. For example, many studies have found that interacting with therapy dogs reduces stress and anxiety and increases positive emotional states in a variety of settings including hospitals, schools and nursing homes (15, 16). In other cases, high public expectations about the healing power of pets are not matched by the results of empirical studies. For instance, while the Human-Animal Bond Research Institute survey found that 86% of pet owners believe pets relieve depression, the majority of studies on pet-ownership and depression do not support these conclusions (17).

Because so many people have extensive personal experiences with dogs, investigators face unique challenges in sharing research results with the public. In their hearts, dog owners *believe* that their canine companions make them feel less depressed, or that dogs feel guilty when they've eliminated indoors or explored the kitchen garbage—even though research might suggest otherwise. In addition, when it comes to animal companions, people much prefer to read a news article in which visits with a therapy dog improved the well-being of a child undergoing chemotherapy than an article about a randomized clinical trial which found no differences between the well-being of children in a therapy dog group and a control group (18). Nor is there likely to be much press coverage devoted to methodological issues such as small effect sizes and inappropriate attributions of causality to the results of correlational studies.

Canine scientists and scholars of human-animal interactions (anthrozoologists) are fortunate that the public is intrinsically interested in our research. We feel that it is critical for investigators to make efforts to communicate the findings of important studies to the public. We caution however, that researchers should not overstate the implications of their findings in press releases and conversations with journalists, despite frequent pressure to do so. These distortions could have a negative impact on misleading the public and misrepresenting the actual findings, a problem that is particularly acute in canine science where well-intentioned pet owners may eagerly adopt practices based on media coverage of scientific studies. The now-established discipline of science communication offers guidance for how best to engage with community and research stakeholders in meaningful ways.

Traditionally, science communication has relied on the knowledge deficit model of communication (19). Directionally one-way, the deficit model operates on the assumption that ignorance is the reason for a lack of community support and application of scientific evidence. Examples where practices have not been updated in response to research findings include dog training methodology (9) and breeding selection for extreme body types, such as brachycephaly in pugs and bulldogs, even though the health and welfare impacts are scientifically well understood (20). Scientists who share their research results thinking that knowledge disseminated—to “educate” the public—is enough to result in different dog care decisions, industry practices or legislation, will generally find this to be ineffective (21). This is because the deficit model overlooks the underlying beliefs, existing attitudes and motivations for current practices. We now recognize that the deficit model is not the most effective way to communicate, engage stakeholders and effect change (22, 23).

Further exploration of the effect of targeted and intentional science communication, informed by human behavior change research, will improve the translation of canine science to meaningful outcomes for dogs and people alike (12). This is important, as many studies in canine science have applied aims designed to inform global policies and the creation of best practices (24, 25). Applied research from the livestock and farming sector suggests that coordinating human behavior change strategies from social and psychological sciences can influence beliefs and attitudes to motivate changes in the ways people behave toward animals, resulting in improved animal welfare (26–28). In the era of attention economics, where scientists are competing for public attention alongside other diverse media, it is vital that the communication of our work is honest, relevant, and effective, to ensure that our field stays on the radar of key stakeholders, funding bodies and change agents.

## Funding

A third key challenge in the future of canine science concerns research funding and a careful balancing of the priorities of scientists and funding agencies. In the last decade, canine science has received considerable support from the pet care sector, as well as human health and defense agencies [e.g., (29)]. Fine and Andersen (30) stress that although funding is still a challenge in human-animal interaction research, there are now more options to be found. In 2008, the Waltham Petcare Science Institute initiated a public-private partnership with the Eunice Kennedy Shriver National Institute of Child Health and Human Development. Over the past decade, this partnership has provided funding for research aimed at measuring the impact of specific Animal-assisted interventions. Since 2014, the Human Animal Bond Research Institute has funded a total of 35 academic research grants investigating the health outcomes of pet ownership and/or human-animal interaction, both for the people and non-human animals involved. Despite clear benefits for enabling research, there remains a limited group of agencies responsible for funding this work. This has potential to constrain the range of topics being studied. In addition, scientists may feel compelled to support the agendas of industry groups, such as those in the pet sector, who often encourage research that will demonstrate the benefits of pets and human-animal interactions.

These constraints were recognized by Wallis Annenberg PetSpace in 2017 when they envisioned their Leadership Institute Program with a mission to promote interdisciplinary scholarship and convene meetings to accelerate research and policy development (https://www.annenbergpetspace.org/about/leadership). This model for engagement inspired the organization to offer two invited retreats (2017, 2020) for a total of 33 experts in the field that provided opportunities for open ended and frank discussion about the nature of human-animal interaction research, and the maturing field of canine science. By providing the space and financial support, plus the opportunity to work together and publish, Annenberg PetSpace provided a way to both illuminate current limitations, and to identify priorities for the future, free of constraints from outside interest groups. These intellectual salons have no specific agenda other than to consider the future of the field and what kinds of questions need to be asked based on what we already know. The results of these two retreats include 14 published refereed papers, plus a suite of collaborations that might otherwise not have happened. We hope that these fellowships and retreats continue and inspire others to support similar initiatives so that scholars across multiple disciplines have the opportunity to experience the transformational exchanges that occur during these programs. The new era of canine science will require diverse funding that is not limited to how dogs can benefit humans, from health, safety and economic perspectives. This change will enable researchers the freedom to further our understanding of dogs and their needs for optimized welfare. In turn, this will allow us to identify how dogs and people can thrive together.

## Looking Ahead

We hope that the publications emerging from these retreats will reach a diverse community of stakeholders, including students, early career researchers, animal welfare and advocacy groups, legislators and policy makers, philanthropies, and traditional agency funders. The goal of these papers is to spark imagination for projects not yet engaged and to help set the agenda for future research that can enhance our understanding of human-dog interactions and identify paths to ensure a future of symbiotic relationships between these species.

The vision of this collective group of scholars includes the goal of establishing studies with dogs as a sustainable and broad-reaching research focus. Although dogs provide many advantages as a “model species” —including their phenotypic diversity, and shared environments and evolutionary history with humans—a research model centered around dogs has many additional benefits. Dogs provide a rich, interactive and sentient model with deep implications for the way scientists approach animal research, and animal welfare. Dogs also increase the accessibility of research, both literally, due to their ubiquity and opportunities for large-scale public participation in research (31, 32), and figuratively, through a body of work with appeal to the broader public.

The field of canine science has much in common with a similar emerging science, that of urban ecology. Humans are historically at the core of the subject material, but non-human elements are often the focus of the study. As such, the work is always culturally embedded, relevant to a variety of stakeholders, and ultimately expected to improve quality of life. The urban ecologists coined a term *Use-Inspired Research* (33) from modifying the existing idea of Pasteur's Quadrant which organizes research questions across the axes of fundamental understanding and considerations of use (34). Both canine research and urban ecology seek fundamental understanding, but also expect to directly apply the knowledge gained to improve outcomes for their subjects and stakeholders.

By including the public in canine science we not only increase the quantity of the data that we can gather, we serve as ambassadors for a new model of responsible animal research. The result increases the value of human-animal interaction research and creates opportunities for the next generation of interdisciplinary scientists. The goal of this collection has been both to highlight specific recent advances in canine science as well as to identify emerging and overarching issues that will shape the future of this field. The multidisciplinary nature of our work with dogs allows scientists to contribute to a robust research agenda, enhancing our understanding of canines and their impact on society. Ultimately, the nexus of our discoveries should have profound effects on reshaping and enriching our relationships with dogs.

## Data Availability Statement

The original contributions presented in the study are included in the article/supplementary material, further inquiries can be directed to the corresponding author/s.

## Author Contributions

All authors listed have made a substantial, direct and intellectual contribution to the work, and approved it for publication.

## Conflict of Interest

The authors declare that the research was conducted in the absence of any commercial or financial relationships that could be construed as a potential conflict of interest.
